# Quantity processing of Chinese numeral classifiers: Distance and congruity effects

**DOI:** 10.1371/journal.pone.0206308

**Published:** 2018-11-07

**Authors:** Ying-Chun Chen, One-Soon Her, Nai-Shing Yen

**Affiliations:** 1 Graduate Institute of Linguistics, National Chengchi University, Taipei, Taiwan; 2 Research Centre for Mind, Brain, and Learning, National Chengchi University, Taipei, Taiwan; 3 Department of Psychology, National Chengchi University, Taipei, Taiwan; Vita-Salute San Raffaele University, ITALY

## Abstract

A numeral classifier is required between a numeral and a noun in Chinese, which comes in two varieties, sortal classifier (C) and mensural classifier (M). A recent linguistic theory suggests that C/Ms carry quantity information, where C and M converge as the multiplicand, with numeral as the multiplier, but C and M diverge in the mathematical values they denote. However, previous empirical studies were sparse and presented inconsistent results. This study aimed to investigate the mathematical function of C/Ms using the number-size task in which participants had to choose from two C/M phrases the one that represents a larger quantity or in a larger font size. If C/M phrases engage quantity processing like numbers, distance and congruity effects should emerge. As expected, participants performed more accurately and faster at comparing two distant stimuli than two proximate ones, indicating that the mathematical values of C/M were represented like a mental number line. Moreover, participants' performance was partially influenced by the irrelevant information from the other dimension, suggesting that the mathematical values of C/Ms and the physical font size interfere with each other. These results corroborated that C/Ms play a role in magnitude cognition.

## Introduction

In a classifier language such as Chinese, an additional element, known as ‘numeral classifier’ or simply ‘classifier’, is required when a noun (N) is quantified by a numeral (Num). Numeral classifiers that can appear in a classifier construction come in two subcategories, sortal classifiers (C) and mensural classifiers (M), which are also often referred to as ‘classifiers’ and ‘measure words’, respectively, among various other terms. In this paper, the overall syntactic category is referred to as ‘numeral classifiers’ or simply ‘classifiers’, abbreviated as ‘C/M’. Table 1 offers examples from Chinese, where *ben* and *ke* are Cs, the former for books and the latter for round objects; *xiang* (box) and *da* (dozen) are Ms.

**Table 1:** Examples of Chinese numeral classifiers.

Sortal Classifier (C)	Mensural Classifier (M)
五 本 雜誌	五 箱 雜誌
*wu ben zazhi*	*wu xiang zazhi*
5 C magazine	5 M-box magazine
‘5 magazines’	‘5 boxes of magazines’
十 顆 蘋果	十 打 蘋果
*shi ke *	*shi da pingguo*
10 C apple	10 M-dozen apple
‘10 apples’	‘10 dozens of apples’

Grammarians had in fact been arguing for a long time whether C and M constitute one or two grammatical categories, until some recent studies that demonstrated convincingly that C and M converge syntactically as one single category, in that they appear in the same structural position and are mutually exclusive [1–3]; yet, C and M diverge semantically, as Cs qualify the noun and contribute no additional semantic information to the noun phrase, while Ms quantify the noun and provide additional information to the noun phrase [4, 5]. The convergence and divergence are further reconciled in Her’s [6] mathematical account, which suggests that the relation between Num and C/M is multiplication. Under this view, C and M converge as the multiplicand, with Num as the multiplier, while they diverge in their respective values: all Cs are equally and necessarily of the numerical value *1*, while an M’s value can be anything that is not necessarily *1*. The precise formulation for the C/M distinction is: [Num X N] = [[Num × X] N], where X = C if and only if X = 1, otherwise X = M [6]. Thus, X, being the single category required between Num and N, is a C if its mathematical value is necessarily 1; otherwise, X is an M. For example, in *shi ke pingguo* (ten C apple), *shi* (ten) and *ke* (C) form a multiplicative unit, i.e., (10×1). Similarly, in *shi da pingguo* (ten M-dozen apple), *shi* (ten) and *da* (M-dozen) also form a multiplicative unit, i.e., (10×12). In brief, C and M both play the role of multiplicand but differ in the sense that C = 1, M ≠ 1.

Her, Chen, & Yen [7] presented a taxonomy of the mathematical values that C/Ms denote based on two dimensions: numerical vs. non-numerical and fixed vs. variable (Table 2). This taxonomy thus also serves to classify C/Ms into five subtypes accordingly. C stands on its own, whose value is numerical and fixed at 1. While M_1_ and M_2_ also both encode numerical values, the former denotes fixed values besides 1 and the latter denotes variable values besides one. Likewise, M_3_ and M_4_ both encode non-numerical values, but the former has fixed values and the latter does not. Thus, C, M_1_, and M_3_ encode fixed values, while M_2_ and M_4_ do not.

**Table 2:** Types of mathematical values denoted by C/Ms.

Numerical	Fixed	**n = 1** e.g., *ben* (本), *ke* (顆), *tiao* (條), *zhi* (隻)	C
		**n = 2** e.g., *dui* (pair 對); **n = 12** e.g., *da* (dozen 打)	M_1_
	Variable	**n > 1** e.g., *pai* (row 排), *bang* (gang 幫), *die* (stack 疊)	M_2_
Non- numerical	Fixed	e.g., *gongjin* (kilogram 公斤), *gongli* (kilometer 公里)	M_3_
	Variable	e.g., *di* (drop 滴), *dai* (bag 袋), *bei* (cup 杯)	M_4_

Her’s [6] theory implies that C/Ms play an important role in denoting mathematical values. However, empirical studies examining quantity processing of C/Ms are scarce and have shown inconsistent results. While the findings by Cui et al. [8] do not support this mathematical view of C/Ms, two more recent studies by Her et al. [7] and Her, Chen, & Yen [9] do support this view.

The study by Cui et al. [8] adopted a semantic distance comparison task using functional magnetic resonance imaging (fMRI) to investigate the neural correlates of quantity processing of Chinese numeral classifiers. Participants were asked to choose between two items the one that was semantically closer to the target item. The study compared the brain activations of processing numeral classifiers with those of processing tool nouns, numbers, and dot arrays and found that processing numeral classifiers and tool nouns induced higher activations in the left inferior frontal gyrus (IFG) and the left middle temporal gyrus (MTG) than numbers and dot arrays. Also, numeral classifiers, tool nouns, numbers, and dot arrays all activated the right IFG, right angular gyrus, right supplementary motor area, right precentral gyrus, left insula, left cerebellum, and bilateral lenticular nucleus. However, the study did not find greater activations for processing numeral classifiers than tool nouns in the right intraparietal sulcus (IPS), which has been shown to represent abstract numerical magnitude [10, 11]. These findings were inconsistent with Her’s [6] mathematical theory of C/Ms, which predicts that processing numeral classifiers would elicit greater brain activities in the right IPS compared with tool nouns, since C/Ms denote mathematical values but tool nouns do not.

Her et al. [7] replicated the semantic distance comparison paradigm in Cui et al. [8] but added the same noun to create minimal pairs of C/M phrases as experimental stimuli. By doing so, they managed to control the semantic attributes of C/Ms, which might have been a confounding factor in Cui et al. [8]. Furthermore, they distinguished the subcategories of C/Ms along the two dimensions in Table 2: numerical type (numerical vs. non-numerical) and mathematical value type (fixed vs. variable) to thoroughly examine whether participants processed different types of C/Ms based on their mathematical values. They found that participants responded more accurately and faster for C/Ms with fixed values than those with variable values regardless of the numerical type. These results suggested that at least some of the Chinese C/Ms denote mathematical values and preliminarily supported Her’s [6] view that C/Ms denote mathematical values.

Her et al. [9] further examined the neural correlates of C/Ms with fixed values by conducting the same task using fMRI. They found that the numeral classifiers induced greater neural activities than tool nouns in the bilateral inferior parietal lobule (IPL), middle frontal gyrus (MFG), right superior frontal gyrus (SFG), and left lingual gyrus. Moreover, they showed that processing numeral classifiers, numbers, dot arrays, and number words elicited conjunct activations in the IPS. These findings again corroborated Her’s [6] mathematical theory of C/Ms by offering neuroimaging evidence implying that mathematical values play a role in the processing of Chinese numeral classifiers.

Given the conflicting findings regarding the quantity processing of C/Ms by previous studies, the aim of the current study was to re-examine the function of mathematical values of C/Ms by investigating whether participants represent them as numbers using another paradigm. Representation of numerical magnitude has shown two robust phenomena: the distance effect and congruity effect [12, 13]. Dehaene, Dehaene-Lambertz, and Cohen [14] proposed that numbers are represented in order like a mental number line in the brain. As the mental representations of adjacent numerals (e.g. 2 and 3) may overlap to some extent, it is harder to discriminate adjacent numerals than distant numerals (e.g. 2 and 7) [15]. Studies also reported that the distance effect held not only for Arabic numerals and number words [16–18] but also angles and lines [19], and dot arrays [20]. This could be interpreted in terms of Walsh’s [21] view that numbers and physical stimuli may overlap and share common cognitive mechanisms in the parietal lobe. Thus, the shared representation of magnitude could cause interference between numerical value and physical size.

Besner and Colheart [13] first reported that reaction times (RT) changed in accordance with the congruity between the numerical value and the physical size. They found that it took less time to compare the digits when the numerical difference between the two digits corresponds to font size difference than when they are incongruent. This demonstrated that although the physical size was irrelevant in the number comparison task, it was hard to ignore and interfered with numerical processing. Henik and Tzelgov [22] further showed that numerical values also interfered with physical sizes. Moreover, congruent pairs facilitated RT compared with neutral trials in which the information of the irrelevant dimension was identical, whereas incongruent pairs took longer than neutral trials.

Given the two aforementioned features of number processing, in the present study we aimed to inspect how mathematical values of C/Ms function by using the number-size comparison task. Such a task is able to test whether C/Ms denote mathematical values and, more importantly, form a multiplication relation with the numerals ahead as Her [6] proposed. We expected to observe that C/M phrases reflect both the distance effect and the congruity effect. First, smaller mathematical value difference of C/M phrases would be harder to differentiate than a larger one, yielding lower accuracy and longer RT. Second, the mathematical value of C/M phrases and their physical size may interfere with each other, that is, the performance of congruent trials would be facilitated and thus be more accurate and faster than neutral trials whereas it would be worsened for incongruent trials, with lower accuracy and longer RT. Third, the mathematical value of C/M phrases may interact with the physical size. For example, it would be more difficult to make a comparison between the C/M phrases with close mathematical value distance plus incongruent physical size than other pairs under the mathematical value task.

## Method

### Participants

Twenty individuals (13 females, 7 males, ages 20–37, mean age = 22.6 ± 3.89 SD) were recruited from National Chengchi University. Participants were right-handed and had normal or corrected-to-normal vision. Their first language is Mandarin. Prior to the experiment, all participants gave written informed consent to the study. All methods were performed in accordance with the ethical principles of the Declaration of Helsinki and were approved by the Research Ethics Committee of National Taiwan University. Participants received NT$100 after finishing the experiment.

### Task

Participants had to make two types of magnitude judgements on a pair of C/M phrases. In the mathematical value task, they had to choose the phrase that represented a larger quantity. On the other hand, in the physical size task, they had to select the phrase that was shown in a larger font size.

### Stimuli and experimental design

We conducted a 2 (task) × 3 (congruity) × 2 (distance) within-subject design. Stimuli were C/M phrases composed of a numeral and a C or M_1_ (i.e., mensural classifiers with fixed numeral values). Including the numeral in the C/M phrase enabled the participants to process the C/M correctly as C/Ms rather than other meanings. To strictly match the word frequency and number of strokes of C/Ms, four C/Ms were selected to be used in the experiment (Table 3). The word frequency indicates the number of occurrence in the database of Modern Mandarin Corpus. It was obtained from the Digital Resources Centre for Global Chinese Language Teaching and Learning by Cheng et al. [23]. The C and M_1_ with matching word frequency and number of strokes were paired for a trial, that is, *sao* (C for boats and ships, n = 1) and *shuang* (pair, M_1_, n = 2) were coupled and *jian* (C for rooms and houses, n = 1) and *dui* (pair, M_1_, n = 2) were put together.

**Table 3:** The four C/Ms used in the experiment.

Type	C/Ms	Word frequency	Number of strokes	Mathematical Value (n)
C	艘 *sao* C-ship	152	15	1
	間 *jian* C-room	387	12	1
M_1_	雙 *shuang* pair	153	18	2
	對 *dui* pair	369	14	2

The numerals in the C/M phrases ranged from one to nine. The distance of the mathematical value between the two phrases was manipulated as either one or three. Regarding distance = |1|, the pairs were in the form of [3C 2M_1_], [5C 3M_1_], [7C 4M_1_], [5C 2M_1_], [7C 3M_1_], and [9C 4M_1_] (Table 4). Take [3C 2M_1_] for example, the quantity of 3C equals to 3 (i.e., 3 × 1) whereas 2M_1_ represents 4 (i.e., 2 × 2). Therefore, the distance between 3C and 2M_1_ is |1|. As for distance = |3|, the pairs were [1C 2M_1_], [3C 3M_1_], [5C 4M_1_], [5C 1M_1_], [7C 2M_1_], and [9C 3M_1_]. The phrases were presented in Kaiu.Tcc (*biao kai ti*) with three font sizes: 16, 19, and 22 (see Table 5 for examples).

**Table 4:** Combinations of C/M phrases for each experimental condition.

	Mathematical Value Task				Physical Size Task			
	Close Distance = |1|		Far Distance = |3|		Close Font sizes: 19, 22		Far Font sizes: 16, 22	
Congruent	3C	2M_1_	1C	2M_1_	1C	2M_1_	3C	2M_1_
	5C	3M_1_	3C	3M_1_	3C	3M_1_	5C	3M_1_
	7C	4M_1_	5C	4M_1_	5C	4M_1_	7C	4M_1_
	5C	2M_1_	5C	1M_1_	5C	1M_1_	5C	2M_1_
	7C	3M_1_	7C	2M_1_	7C	2M_1_	7C	3M_1_
	9C	4M_1_	9C	3M_1_	9C	3M_1_	9C	4M_1_
Neutral	3C	2M_1_	1C	2M_1_	4C	4C	4C	4C
	5C	3M_1_	3C	3M_1_	4M_1_	4M_1_	4M_1_	4M_1_
	7C	4M_1_	5C	4M_1_				
	5C	2M_1_	5C	1M_1_				
	7C	3M_1_	7C	2M_1_				
	9C	4M_1_	9C	3M_1_				
Incongruent	3C	2M_1_	1C	2M_1_	1C	2M_1_	3C	2M_1_
	5C	3M_1_	3C	3M_1_	3C	3M_1_	5C	3M_1_
	7C	4M_1_	5C	4M_1_	5C	4M_1_	7C	4M_1_
	5C	2M_1_	5C	1M_1_	5C	1M_1_	5C	2M_1_
	7C	3M_1_	7C	2M_1_	7C	2M_1_	7C	3M_1_
	9C	4M_1_	9C	3M_1_	9C	3M_1_	9C	4M_1_

C and M_1_ represents either the pair of *sao* and *shuang* or the pair of *jian* and *dui* with matching word frequency and number of strokes. Note that the font sizes in this table only resembled the experimental conditions relatively and were not exactly the same sizes as they were in the experiment.

**Table 5:** Sample set of experimental stimuli for each condition.

	Mathematical Value Task				Physical Size Task			
	Close Distance = |1|		Far Distance = |3|		Close Font sizes: 19, 22		Far Font sizes: 16, 22	
Congruent	三艘	兩雙	一艘	兩雙	一艘	兩雙	三艘	兩雙
	3 × 1	2 × 2	1 × 1	2 × 2	1 × 1	2 × 2	3 × 1	2 × 2
Neutral	三艘	兩雙	一艘	兩雙	四艘	四艘	四艘	四艘
	3 × 1	2 × 2	1 × 1	2 × 2	4 × 1	4 × 1	4 × 1	4 × 1
Incongruent	三艘	兩雙	一艘	兩雙	一艘	兩雙	三艘	兩雙
	3 × 1	2 × 2	1 × 1	2 × 2	1 × 1	2 × 2	3 × 1	2 × 2

The examples demonstrated a set of trials using *sao* (C-_ship_, n = 1) and *shuang* (pair, M_1_, n = 2) for each condition in this study. The calculation of the mathematical values of C/M phrases were formulised beneath each example. Note that the font sizes in this table only resembled the experimental conditions relatively and were not exactly the same sizes as they were in the experiment.

In order to balance the stimuli while reducing stimulus complexity, we followed the number-size interference paradigm by Kaufmann et al. [24]. To be more specific, in the current experiment, the small mathematical value distance (distance = 1) was always combined with a large font size distance (16 and 22). The large mathematical value distance (distance = 3) was always combined with a small font size distance (19 and 22). By doing so, we could present the physically identical stimuli in both tasks.

The mathematical value difference may be congruent (e.g., 3C 2M_1_), incongruent (e.g., 3C 2M_1_), or non-relevant with the font size difference (Tables 4 and 5). For non-relevant trials (neutral condition) in the mathematical value task, the font size was always 19 (e.g. [3C 2M_1_]. For neutral trials in the physical size task, half of the stimuli were as [4C 4C] and the other half were in the form of [4M_1_ 4M_1_]. Each pair of the stimuli was presented twice, with the correct answer once on the left and once on the right, in two different blocks of the same task.

There were four blocks in the experiment, two of which were mathematical value tasks and the other two were font size tasks. Each type of task alternated with one another. The order of the tasks was counter-balanced. That is, half of the participants performed the mathematical value task first and the other half did the font size task first. Each block was composed of 72 trials (24 congruent trials, 24 incongruent trials, and 24 neutral trials). Consequently, there were 288 trials in total.

The order of the trials within a block was randomised. The experimental programme was written with E-prime 2.0 (Psychology Software Tools, Sharpsburg, PA, USA). The phrase shown on the left was set as 42% on the x-axis while the phrase on the right was at 58% on the x-axis.

### Procedure

**Fig 1:**
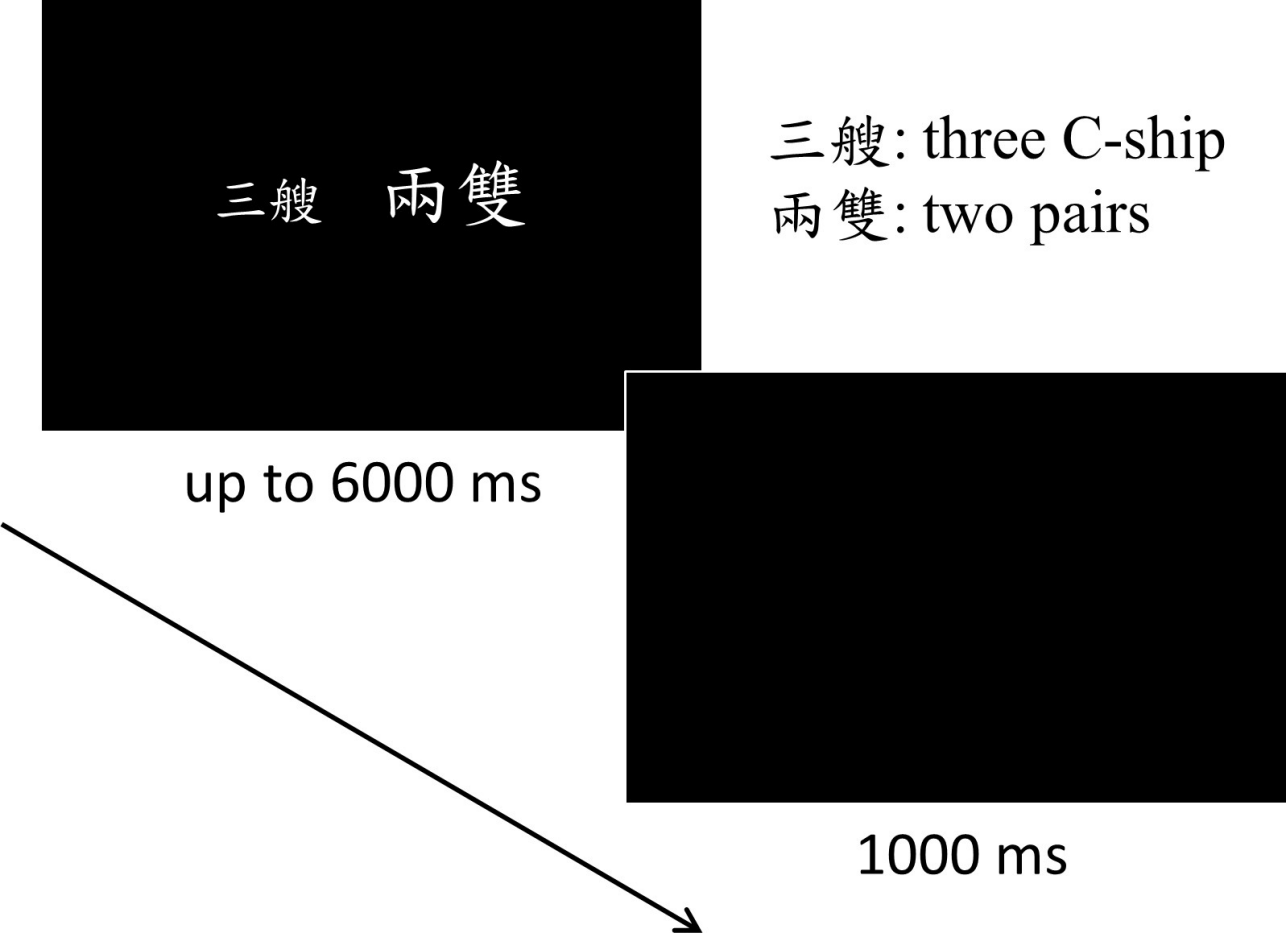
The experimental procedure of a trial. There were two tasks in the current experiment: the mathematical value task and the physical size task. Participants had up to 6000 ms to choose the phrase that represented a larger quantity or the one that was shown in a larger font size according to the task instructions. The inter-trial interval was 1000 ms. The stimuli shown here were an example of the congruent condition (i.e., 3C 2M_1_) with close distance (distance = 1) in the mathematical value task. Note that the stimuli in this figure were not exactly the same size as used in the experiment. They were enlarged proportionately to better demonstrate the experimental conditions.

There were twelve practice trials to ensure that participants fully understood the task. At the beginning of each block, the screen showed the task and the instructions. Then, a fixation cross appeared for 1000 ms. Each trial began with the presentation of the stimuli for up to 6000 ms (Fig 1). Then a blank screen was presented for 1000 ms. No feedback was given to the participants. The responses and RT were recorded.

### Data analysis

Among the twenty participants, one participant was excluded from the data analysis because of data loss. Mean RT were calculated from correct trials only. The responses, RT, and standardised performance scores were analysed in a three-way (Task × Congruity × Distance) repeated measures ANOVA. Furthermore, in order to combine accuracy and RT in a single measure, we also analysed a standardised performance score as an index of overall performance [25]. The standardised performance score was obtained by first subtracting the Z score of RT from the Z score of accuracy and then dividing this number by two. Higher scores (i.e., higher accuracy and shorter RT) indicate better overall performance.

In response to one of the reviewers’ valuable comment, which questioned whether the congruity effect was caused by the numerals alone as opposed to the multiplicative products from numerals and classifiers. Two additional three-way 2 × 2 × 2 (Congruity of numerals × Congruity of products × Distance) repeated measures ANOVAs were implemented for the mathematical value task and the physical size task respectively. The congruity of numerals refers to the consistency between the direction of the pair of numerals and the direction of the pair of physical font sizes. Take [3C 2M_1_] for instance, the congruity of numerals (3 > 2) is incongruent with the sizes (smaller on the left) whereas the congruity of products (3 < 4) is congruent with the physical font sizes. By including the congruity of numerals as an independent variable in the additional analyses, its influences on accuracy and RT as well as interaction with other factors could be revealed. It is worth noting that there was no neutral condition in these analyses because of the lack of suitable trials.

SPSS 21 (IBM, Armonk, NY, USA) was used for the statistical analysis with the α value set at .05. Post-hoc analyses of the simple main effects were made by means of t-tests applying Bonferroni’s correction for multiple comparisons.

## Results

**Fig 2:**
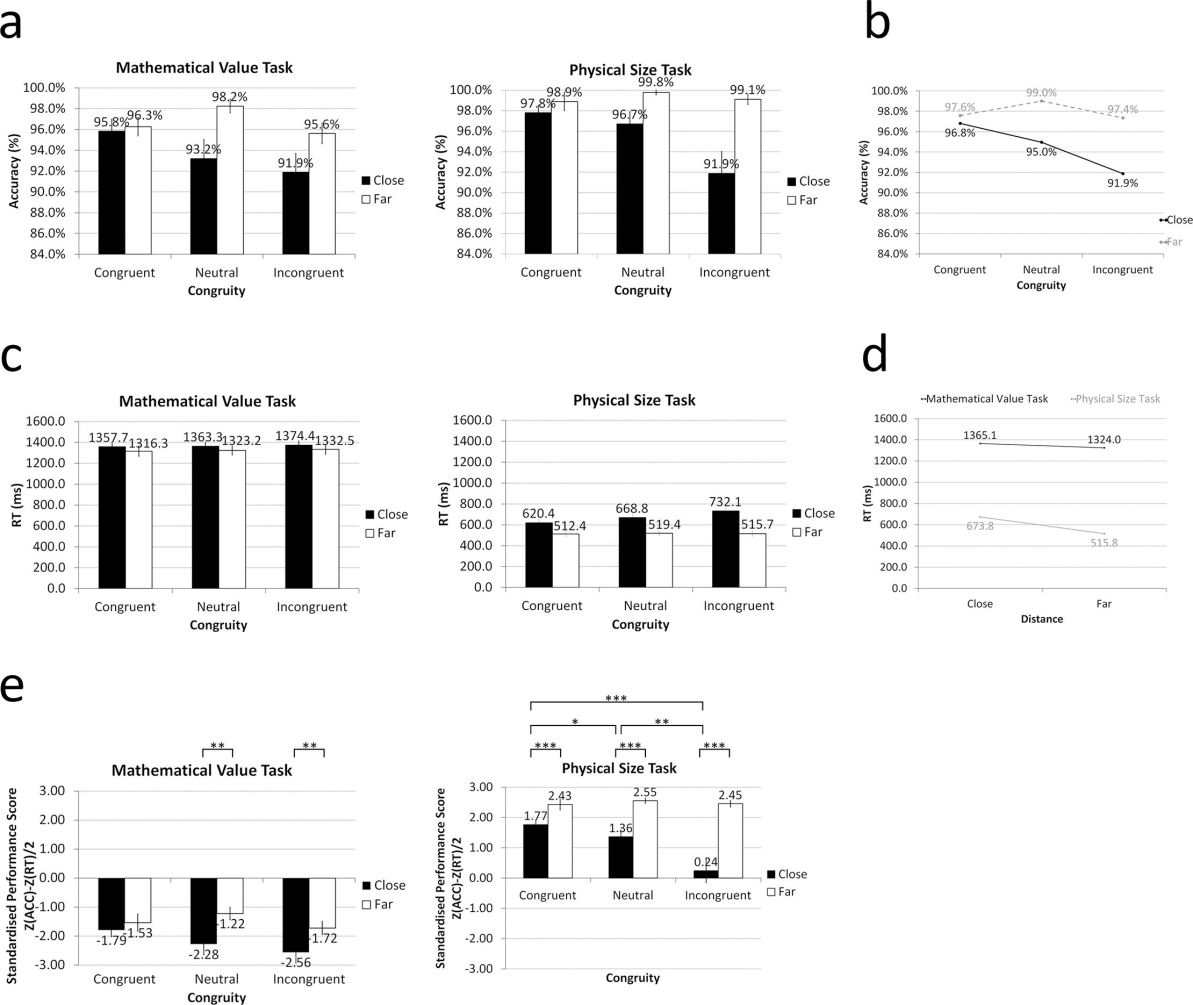
(a) Mean accuracy, (b) interaction of accuracy between congruity and distance, (c) mean RT, (d) interaction of RT between task and distance, and (e) mean standardised performance score. Error bars indicate standard error of the mean (SEM). Asterisk * marks *p* ≤ .05, ** marks *p* ≤ .01, *** marks *p* ≤ .001.

**Fig 3:**
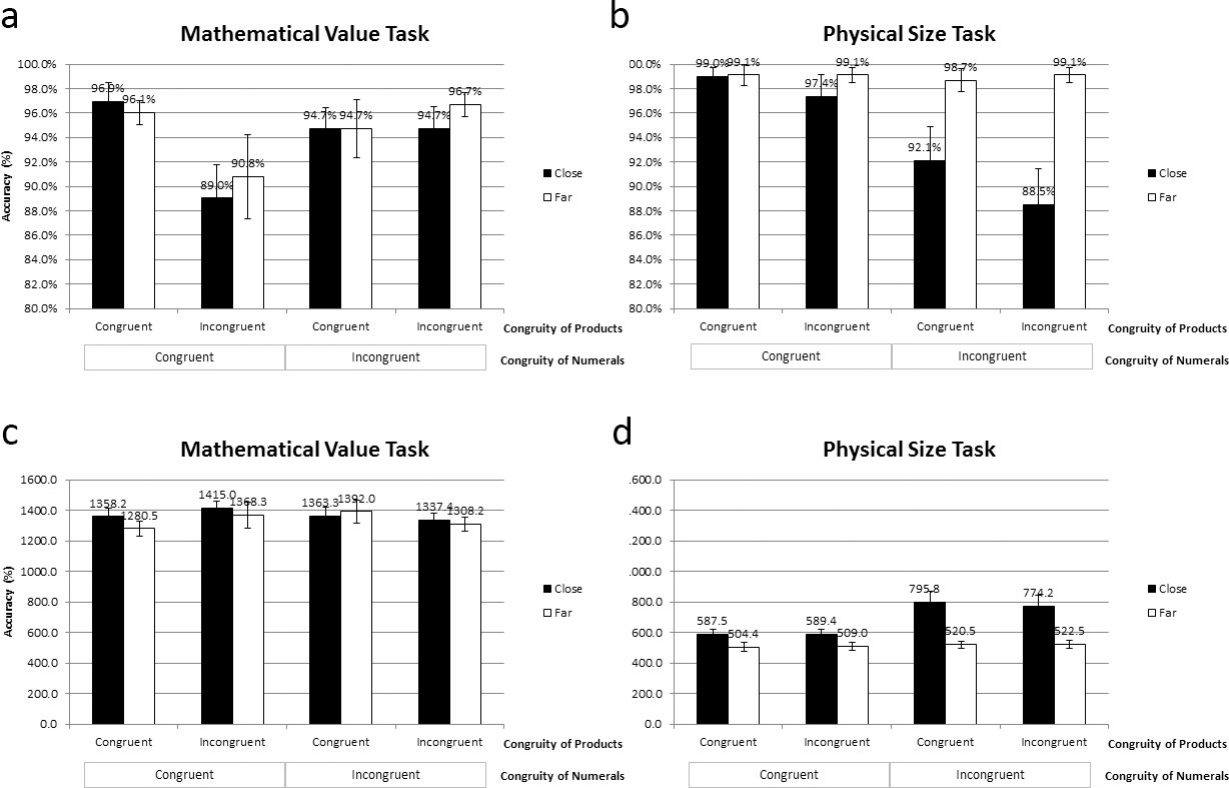
**Mean accuracy in the (a) mathematical value task, and the (b) physical size task; mean RT in the (c) mathematical value task, and the (d) physical size task.** Error bars indicate SEM. Asterisk * marks *p* ≤ .05, ** marks *p* ≤ .01, *** marks *p* ≤ .001.

The mean (and standard error of the mean, SEM) of accuracy, RT, and standardised performance score are shown in Fig 2, Fig 3, and S1 Dataset.

### The interaction between task, congruity, and distance in both tasks

#### Accuracy

There was a significant main effect of task, such that the accuracy of the physical size task (97.4%) was significantly higher than that of the mathematical value task (95.3%), *F*_(1, 18)_ = 5.109, *p* < .05, *η*^2^ = .221 (Fig 2A). The main effect of congruity was also significant, such that the accuracy of incongruent trials was the lowest compared to congruent and neutral trials (94.8% vs. 97.3% vs. 97.1%; respectively), *F*_(2, 36)_ = 5.373, *p* < .01, *η*^2^ = .230. Moreover, there was a significant main effect of distance, such that the accuracy of close distance (94.7%) was significantly lower than that of far distance (98.1%), *F*_(1, 18)_ = 21.350, *p* < .001, *η*^2^ = .543. No significant interaction effect between the task and congruity (*F*_(2,36)_ = .246, *p* = .783, *η*^2^ = .014), or, task and distance (*F*_(1, 18)_ = .298, *p* = .592, *η*^2^ = .016) was found. However, there was a significant interaction between the congruity and distance, *F*_(2, 36)_ = 6.555, *p* < .01, *η*^2^ = .267 (Fig 2B). Lastly, the three-way interaction between the task, congruity, and distance was not significant, *F*_(2, 36)_ = 2.192, *p* = .126, *η*^2^ = .109.

#### Reaction times

There was a significant main effect of task, such that the RT in the mathematical value task (1344.57 ms) were significantly longer than in the physical size task (594.82 ms), *F*_(1, 18)_ = 262.907, *p* < .001, *η*^2^ = .936 (Fig 2C). Nevertheless, the main effect of congruity was not significant, *F*_(2, 36)_ = 2.734, *p* = .078, *η*^2^ = .132. The main effect of distance was significant, such that the RT of close distance (1019.46 ms) were longer than those of far distance (919.93 ms), *F*_(1, 18)_ = 43.713, *p* < .001, *η*^2^ = .708. Furthermore, the distance effect was larger in the physical size task, leading to a significant interaction between the task and distance, *F*_(1, 18)_ = 17.149, *p* < .01, *η*^2^ = .488 (Fig 2D). No significant interaction effect between the task and congruity (*F*_(2, 36)_ = .840, *p* = .440, *η*^2^ = .045), or, congruity and distance (*F*_(2, 36)_ = 2.363, *p* = .109, *η*^2^ = .116) was found. Lastly, there was no significant three-way interaction effect between the task, congruity, and distance, *F*_(2, 36)_ = 2.015, *p* = .148, *η*^2^ = .101 (Fig 2B).

#### Standardised performance score

There was a significant main effect of task, such that the standardised performance score in the physical size task was significantly higher than in the mathematical value task, *F*_(1, 18)_ = 184.124, *p* < .001, *η*^2^ = .911 (Fig 2E). The lower score for incongruent compared with congruent and neutral trials (-.395 vs. .217 vs. .105; respectively) led to a significant main effect of congruity, *F*_(2, 36)_ = 7.480, *p* < .01, *η*^2^ = .294. Moreover, the main effect of distance was also significant, such that the standardised performance score for close distance (-.542) was lower than that for longer distance (.494), *F*_(1, 18)_ = 62.268, *p* < .001, *η*^2^ = .776. No significant interaction effect between the task and congruity (*F*_(2, 36)_ = .620, *p* = .544, *η*^2^ = .033) was found. However, there was a significant interaction between the task and distance (*F*_(2, 36)_ = 6.643, *p* < .05, *η*^2^ = .270), and congruity and distance (*F*_(2, 36)_ = 11.048, *p* < .001, *η*^2^ = .380).There was also a significant three-way interaction effect between the task, congruity, and distance, *F*_(2, 36)_ = 4.280, *p* < .05, *η*^2^ = .192. The distance effect was only absent for the congruent condition in the mathematical value task. Furthermore, the congruity effect was significant for close distance rather than far distance in the physical size task.

### The interaction between congruity of numerals, congruity of products, and distance in each of the tasks

#### Accuracy

In terms of the mathematical value task, there was no significant main effect of the congruity of numerals (*F*_(1, 18)_ = 2.749, *p* = .115, *η*^2^ = .132), no main effect of congruity of products (*F*_(1, 18)_ = 2.747, *p* = .115, *η*^2^ = .132), nor distance (*F*_(1, 18)_ = .349, *p* = .562, *η*^2^ = .019) (Fig 3A). However, the congruity effect of products was larger when the congruity of numerals were congruent rather than incongruent, leading to a significant interaction between the congruity of numerals and the congruity of products (*F*_(1, 18)_ = 4.977, *p* < .05, *η*^2^ = .217). No significant interaction effect between the congruity of numerals and distance (*F*_(1, 18)_ = .039, *p* = .846, *η*^2^ = .002), or, the congruity and distance (*F*_(1, 18)_ = 1.703, *p* = .208, *η*^2^ = .086) was found. Lastly, there was no significant three-way interaction effect between the congruity of numerals, congruity of products, and distance, *F*_(1, 18)_ = .002, *p* = .968, *η*^2^ < .001.

Regarding the physical size task, there was a significant main effect of the congruity of numerals, such that the accuracy was higher when the congruity between numerals and font sizes was congruent (98.7%) than incongruent (94.7%), *F*_(1, 18)_ = 22.511, *p* < .001, *η*^2^ = .556 (Fig 3B). In addition, the main effect of distance was also significant, such that the accuracy of close distance (94.3%) was significantly lower than that of far distance (99.0%), *F*_(1, 18)_ = 22.024, *p* < .001, *η*^2^ = .550. However, the main effect of congruity of products was not significant, *F*_(1, 18)_ = 1.352, *p* = .260, *η*^2^ = .070. Furthermore, the congruity effect of numerals was larger when the distance was close, resulting in a significant interaction between the congruity of numerals and distance, *F*_(1, 18)_ = 20.783, *p* < .001, *η*^2^ = .536. No significant interaction effect between the congruity of numerals and congruity of products (*F*_(1, 18)_ = .115, *p* = .739, *η*^2^ = .006), or, congruity of products and distance (*F*_(1, 18)_ = 1.398, *p* = .252, *η*^2^ = .072) was found. Lastly, there was no significant three-way interaction effect between the congruity of numerals, congruity of products, and distance, *F*_(1, 18)_ = .278, *p* = .604, *η*^2^ = .015.

#### Reaction times

Concerning the mathematical value task, there was no significant main effect of the congruity of numerals (*F*_(1, 18)_ = .020, *p* = .890, *η*^2^ = .001), no main effect of congruity of products (*F*_(1, 18)_ = .058, *p* = .813, *η*^2^ = .003), nor distance (*F*_(1, 18)_ = 1.861, *p* = .189, *η*^2^ = .094) (Fig 3C). Moreover, No significant interaction effect between the congruity of numerals and congruity of products (*F*_(1, 18)_ = 3.636, *p* = .073, *η*^2^ = .168), congruity of number and distance (*F*_(1, 18)_ = .981, *p* = .335, *η*^2^ = .052), or, congruity of products and distance (*F*_(1, 18)_ = .039, *p* = .846, *η*^2^ = .002) was found. Lastly, there was no significant three-way interaction effect between the congruity of numerals, congruity of products, and distance, *F*_(1, 18)_ = 1.678, *p* = .212, *η*^2^ = .085.

In respect of the physical size task, the main effect of the congruity of products was not significant, *F*_(1, 18)_ = .026, *p* = .875, *η*^2^ = .001 (Fig 3D). Nevertheless, there was a significant main effect of the congruity of numerals, such that the RT with congruent congruity of numerals were shorter than that with incongruent congruity of numerals, *F*_(1, 18)_ = 16.281, *p* < .001, *η*^2^ = .475. Furthermore, the main effect of distance was also significant, such that the RT of close distance (686.720 ms) were longer than those of far distance (514.112 ms), *F*_(1, 18)_ = 35.994, *p* < .001, *η*^2^ = .667. In addition, the distance effect was larger when the congruity of numerals was incongruent, which lead to a significant interaction effect between the congruity of numerals and distance was significant, *F*_(1, 18)_ = 14.634, *p* < .001, *η*^2^ = .448. However, no significant interaction effect between the congruity of numerals and congruity of products (*F*_(1, 18)_ = .077, *p* = .785, *η*^2^ = .004), or, congruity of products and distance (*F*_(1, 18)_ = .109, *p* = .745, *η*^2^ = .006) was found. Last but not least, the three-way interaction between the congruity of numerals, congruity of products, and distance was not significant, (*F*_(1, 18)_ = .060, *p* = .809, *η*^2^ = .003).

## Discussion

In the present experiment, we examined the role of mathematical values of C/Ms using the number-size comparison task. In terms of accuracy, first, we observed that participants responded more accurately when comparing the physical size of the C/M phrases than the mathematical values of C/M phrases. Moreover, as predicted, we found that participants answered more accurately when making a judgment between stimuli with farther distance (i.e., either mathematical value or physical size) regardless of the tasks. Furthermore, incongruent trials led to the lowest accuracy rate, i.e., lower than neutral and congruent trials, in both tasks. Last but not least, distance and congruity showed an interaction effect. Participants were more easily affected by the information from the irrelevant dimension (size/value) when comparing the stimuli with closer value/size distance. To be more specific, when participants made a judgment between a pair with small numerical/physical distance, congruent pairs were answered more accurately whereas answers to incongruent pairs were more error prone. On the other hand, congruity did not have such an impact on stimuli with far value/size distance.

Moreover, participants spent longer time comparing the mathematical values of C/Ms than physical size. In addition, similar to the accuracy rate, distance effect of RT was observed. Although the distance effect was larger in the physical size task, mathematical value task also revealed the same trend, i.e., the closer the distance, the longer the RT. However, congruity did not have a significant influence on RT. Though not significant, physical size task exhibited the pattern of facilitation for congruent trials (shorter RT than neutral trials) and interference for incongruent trials (longer RT than neutral trials) when comparing stimuli with close physical sizes. This suggests that the mathematical value of C/Ms may have some interference effect on the RT of physical size. Nonetheless, it can be seen that the mean RT for far distance in the physical size task was extremely short and almost the same among the three conditions of congruity. This may indicate that comparing physical sizes with far distance was too easy to respond to before the irrelevant information (i.e., mathematical value of C/Ms) could interfere.

Since accuracy and RT did not display the same pattern, we also looked into the standardised performance score, which is an index of overall performance. The results were very similar to the findings of accuracy. First, participants performed better in the physical size task compared to the mathematical task. Second, except for the congruent trials in the mathematical value task, distance effect emerged in all other conditions. This is probably because that the consistent physical sizes facilitated the performance for comparing C/Ms with close distance in the mathematical value task, making it as comparatively easy as comparing C/Ms with far distance and resulting in non-significant difference between them. As found in RT results, congruity effect was not as apparent in the mathematical value task as in the physical size task. Nonetheless, the congruity effect was remarkable when comparing physical sizes with close distance. This demonstrated that the mathematical value of C/Ms impeded the process of comparing similar physical sizes.

One may argue that the effects observed in this study were caused by the numerals alone instead of the multiplicative product from numerals and classifiers. Thus, in order to examine specifically whether the distance of the numerals alone had an impact on accuracy and RT in the mathematical value task, we performed a 3 (congruity) × 2 (distance of product) × 2 (distance of numerals) ANOVA. Distance of numerals did not have a significant effect on either accuracy (*F*_(1, 18)_ = 1.382, *p* = .255, *η*^2^ = .071) or RT (*F*_(1, 18)_ = .430, *p* = .520, *η*^2^ = .023). However, the distance of products from numerals and classifiers showed significant effect on both accuracy (*F*_(1, 18)_ = 7.635, *p* < .05, *η*^2^ = .298) and RT (*F*_(1, 18)_ = 6.207, *p* < .05, *η*^2^ = .256). Taken together, although the stimuli included not only classifiers but also numerals, the distance effect stemmed from the multiplicative product from numerals and classifiers rather than the numerals alone.

In the present experiment, only around half of the trials had inconsistent numerical directions between the pair of numerals and the pair of C/Ms. Take [3C 2M_1_] for example, the direction of the value of the products, i.e. [(3 × 1) < (2 × 2)] is different from the direction of the value of numerals alone, i.e. [3 > 2]. The other trials had consistent directions between the pair of numerals and the pair of C/Ms. For these consistent trials, the congruity of numerals and the congruity of products could not be discriminated. Therefore, one may reasonably suspect that the congruity effect observed in this study was not caused purely by the congruity of products from numerals and C/Ms and that the congruity of numerals may have played a role. Subsequently, in order to examine the role of congruity of numerals in the experiment, we added the second set of analyses. Notably, some neutral trials were not suitable and were thus excluded.

Results showed that, although the congruity effect of numerals was not significant on accuracy nor on RT in the mathematical value task, it was significant on both accuracy and RT in the physical size task. To be more specific, participants answered less accurately and spent longer time choosing between two C/M phrases with different font sizes when the congruity of numerals was incongruent than congruent. This showed that the value of numerals intervened with the font size of C/M phrases. According to Her's [6] theory, numerals and C/Ms form a multiplicative unit, in which numerals play the role of multiplier and C/Ms as multiplicand. That is to say, when participants had to compare the quantity of the two C/M phrases, i.e. [Num C/M], the value of numerals alone must be taken into account first. The literature has shown robust congruity effect between numerical values and physical sizes [13, 22]. Taken together, our results again showed that the congruity of numerals played an important role in the physical size task in this experiment.

However, congruity of products did not have a significant main effect on accuracy nor RT in each task. Nonetheless, there was a significant interaction between the congruity of products and the congruity of numerals in the mathematical value task. More precisely, participants answered more accurately judging the quantity of C/M phrases when the congruity of products was congruent compared with incongruent under the congruent numeral condition. However, when the congruity of numerals was incongruent, congruity effect of products on accuracy was not significant. It is also worth noting that the congruity of products also showed a pattern in which participants answered more accurately for congruent trials than incongruent trials when the distance was close regardless of the congruity of numerals in the physical size task. Although not significant, these patterns were consistent with the findings of the first analysis, which showed that participants answered more accurately when the mathematical values of C/M phrases were congruent with the font sizes than when they were incongruent in both tasks. Admittedly, the congruity effect of products diminished when the congruity of numerals had been taken into account, suggesting that the congruity of numerals may have been a confounding factor in the experiment. Therefore, the results of the first analysis should be interpreted with caution. It is also important to be aware that the first analysis incorporated all trials including neutral trials whereas the second set of analyses only looked into a part of the data.

One limitation of this experiment was the selection of stimuli. Although we created stimuli with both consistent and inconsistent directions of values between the pair of numerals and the pair of C/Ms, the congruity effect of numerals did not average out. While we combined C/Ms with a variety of numerals to strictly manipulate the distance of each pair of C/M phrases, incorporating several numerals also made the observed congruity effect of products hard to interpret because the components of products, i.e. numerals, intervened with the physical sizes as well. Originally, we aimed to examine whether processing C/M phrases manifests two robust phenomena of quantity processing, i.e. congruity effect [13, 22, 24] and distance effect [12, 16–20]. Nonetheless, our findings suggested that our design can be improved in detecting solid congruity effect of products. Future studies should examine these effects in separate experiments using different sets of stimuli to prevent potential confounding factors that numerals may produce. Moreover, we only chose two types of C/Ms (i.e. C: *sao*, *jian*, and M_1_: *shuang*, *dui*) as the experimental stimuli so that we could manipulate the distance and control the word frequency and number of strokes carefully. The quantity processing of other sub-types of C/Ms such as M_3_ and M_4_ remains unclear and needs further investigation.

In all, if C/Ms denote mathematical values and form a multiplicative relation with Num, we should be able to observe typical features of magnitude processing, i.e., distance effect [12, 16–20] and congruity effect of products [13, 22, 24]. Participants indeed performed better at comparing the two distant stimuli than the proximate ones. This was consistent with the view that mental representation of adjacent numbers overlap to some degree. It is thus more difficult to distinguish them than remote numbers [15]. Moreover, participants' performance was affected by the irrelevant information from the other dimension, suggesting that the mathematical values of C/Ms and physical size interfere with each other mutually. This was in line with Walsh [21], who suggested that there is a common coding system of numerical and physical dimensions. Moreover, accuracy rates showed interaction between congruity and distance, such that the congruity effect was larger when participants compared stimuli with closer distance. Specifically, when the task on hand was at a higher difficulty level, influence from the irrelevant dimension was crucial. Performance of congruent trials may be facilitated whereas that of incongruent trials could be worsened. However, the congruity of products may be actually smaller if the congruity of numerals was considered.

To summarise, these findings partially supported Her's [6] theory, which indicated that C and M converge as the multiplicand––with Num as the multiplier––and diverge with different mathematical values, i.e., C = 1, M ≠ 1. Significant distance effect corroborated the claim that the relation between Num and C/M was multiplication because the distance between the C/M phrases was manipulated as Her's [6] theory predicted.

In conclusion, this study contributed to the literature by offering empirical evidence of quantity processing of C/Ms. While Cui et al. [8] reported that the neural correlates of processing numeral classifiers was similar to that of tool nouns instead of that of numbers and dot arrays using the semantic distance comparison task, Her et al. [7, 9] followed the same paradigm but showed different behavioural and neuroimaging results, which suggested that C/Ms represent quantity. Moreover, our results also showed that the mathematical value of C/M and physical size may interfere with each other to some extent, suggesting that these two dimensions may share common cognitive mechanisms [21]. This is consistent with the previous neuroimaging finding that showed processing numeral classifiers, numbers, dot arrays, and number words elicited conjunct activations in the IPS, which has been shown to engage in magnitude representation [9–11]. Last but not least, results from the current experiment not only demonstrated with a different task that C/Ms denote mathematical values (C = 1, M ≠ 1) but also verified that the relation between Num and C/M is multiplication. Because previous studies only used 1 as Num in C/M phrases [7–9], the relation between Num and C/M remained unknown. However, our experiment found the distance effect with a range of combinations of Num and C/Ms as stimuli, verifying that Num and C/M form a multiplicative relation [6]. In sum, the linguistic system of C/M interacts with magnitude cognition.

## Supporting information

S1 Dataset The mean accuracy, RT, and standardised performance score for each participant in this study. (DOCX)
