# Supplementary material for: Quantity processing of Chinese numeral classifiers: Distance and congruity effects
Source: PLoS One. 2018 Nov 7;13(11):e0206308. doi: 10.1371/journal.pone.0206308 (PMC6221336; doi:10.1371/journal.pone.0206308)
Supplement: S1 Dataset — (DOCX) [file pone.0206308.s001.docx]

|  | Mathematical Value Task | | | | | | Physical Size Task | | | | | |
| --- | --- | --- | --- | --- | --- | --- | --- | --- | --- | --- | --- | --- |
|  | Congruent | | Incongruent | | Neutral | | Congruent | | Incongruent | | Neutral | |
|  | Close | Far | Close | Far | Close | Far | Close | Far | Close | Far | Close | Far |
| ACC | ValueConClo | ValueConFar | ValueInconClo | ValueInconFar | ValueNeuClo | ValueNeuFar | SizeConClo | SizeConFar | SizeInconClo | SizeInconFar | SizeNeuClo | SizeNeuFar |
| 1 | 100% | 100% | 88% | 88% | 96% | 92% | 100% | 100% | 92% | 100% | 96% | 100% |
| 2 | 96% | 100% | 75% | 92% | 96% | 100% | 92% | 83% | 58% | 92% | 83% | 100% |
| 3 | 92% | 92% | 96% | 92% | 88% | 96% | 92% | 100% | 88% | 100% | 83% | 100% |
| 4 | 100% | 100% | 100% | 96% | 96% | 96% | 100% | 100% | 96% | 100% | 96% | 100% |
| 5 | 83% | 88% | 92% | 92% | 96% | 100% | 92% | 100% | 88% | 96% | 96% | 100% |
| 6 | 100% | 100% | 92% | 100% | 92% | 100% | 100% | 100% | 100% | 100% | 100% | 100% |
| 7 | 100% | 96% | 100% | 100% | 100% | 100% | 100% | 100% | 96% | 100% | 96% | 100% |
| 8 | 96% | 92% | 88% | 96% | 96% | 92% | 100% | 100% | 92% | 100% | 100% | 100% |
| 9 | 100% | 100% | 96% | 100% | 92% | 96% | 96% | 96% | 100% | 100% | 92% | 100% |
| 10 | 100% | 96% | 100% | 100% | 100% | 100% | 100% | 100% | 100% | 100% | 100% | 100% |
| 11 | 92% | 92% | 96% | 92% | 88% | 100% | 100% | 100% | 88% | 100% | 100% | 100% |
| 12 | 88% | 92% | 71% | 88% | 67% | 100% | 96% | 100% | 92% | 100% | 100% | 100% |
| 13 | 100% | 96% | 96% | 100% | 92% | 96% | 100% | 100% | 92% | 96% | 96% | 96% |
| 14 | 100% | 100% | 96% | 100% | 83% | 100% | 100% | 100% | 100% | 100% | 100% | 100% |
| 15 | 96% | 100% | 96% | 96% | 100% | 100% | 100% | 100% | 92% | 100% | 100% | 100% |
| 16 | 92% | 96% | 96% | 96% | 96% | 100% | 100% | 100% | 88% | 100% | 100% | 100% |
| 17 | 92% | 96% | 88% | 96% | 100% | 100% | 100% | 100% | 92% | 100% | 100% | 100% |
| 18 | 100% | 96% | 88% | 100% | 100% | 100% | 96% | 100% | 100% | 100% | 100% | 100% |
| 19 | 96% | 100% | 96% | 96% | 96% | 100% | 96% | 100% | 96% | 100% | 100% | 100% |
| Mean | 95.8% | 96.3% | 91.9% | 95.6% | 93.2% | 98.2% | 97.8% | 98.9% | 91.9% | 99.1% | 96.7% | 99.8% |
| SD | 5.0% | 3.9% | 7.9% | 4.3% | 8.0% | 2.9% | 3.2% | 3.9% | 9.4% | 2.2% | 5.3% | 1.0% |
| SEM | 1.1% | 0.9% | 1.8% | 1.0% | 1.8% | 0.7% | 0.7% | 0.9% | 2.1% | 0.5% | 1.2% | 0.2% |

| RT | ValueConClo | ValueConFar | ValueInconClo | ValueInconFar | ValueNeuClo | ValueNeuFar | SizeConClo | SizeConFar | SizeInconClo | SizeInconFar | SizeNeuClo | SizeNeuFar |
| --- | --- | --- | --- | --- | --- | --- | --- | --- | --- | --- | --- | --- |
| 1 | 1270.63 | 1414.50 | 1595.14 | 1341.00 | 1239.39 | 1170.73 | 517.17 | 506.88 | 629.68 | 540.38 | 587.91 | 509.42 |
| 2 | 1134.43 | 1045.71 | 1343.78 | 1156.55 | 1260.09 | 1097.54 | 546.00 | 461.55 | 522.71 | 403.36 | 547.15 | 428.42 |
| 3 | 1531.18 | 1457.77 | 1568.57 | 1591.32 | 1624.24 | 1710.70 | 521.77 | 415.67 | 532.24 | 422.92 | 573.85 | 497.88 |
| 4 | 1386.33 | 1306.21 | 1245.67 | 1178.39 | 1287.17 | 1148.61 | 1074.54 | 697.67 | 1657.22 | 750.50 | 1072.74 | 689.21 |
| 5 | 1276.20 | 1443.76 | 1416.68 | 1380.55 | 1357.61 | 1236.29 | 477.41 | 475.92 | 737.05 | 493.48 | 589.04 | 518.50 |
| 6 | 1231.46 | 1232.00 | 1330.82 | 1453.67 | 1265.73 | 1286.83 | 752.79 | 486.25 | 652.58 | 509.96 | 797.13 | 517.63 |
| 7 | 1811.21 | 1911.91 | 1689.25 | 1413.25 | 1491.33 | 1571.58 | 790.29 | 622.04 | 823.65 | 588.92 | 764.83 | 547.21 |
| 8 | 1388.43 | 1297.14 | 1350.90 | 1443.52 | 1629.00 | 1328.32 | 695.96 | 799.42 | 1031.41 | 729.38 | 864.33 | 757.42 |
| 9 | 1310.17 | 1204.75 | 1170.26 | 1124.83 | 1338.41 | 1265.22 | 608.17 | 488.70 | 821.63 | 463.50 | 624.41 | 476.08 |
| 10 | 1710.92 | 1505.30 | 1418.08 | 1651.04 | 1581.79 | 1488.67 | 636.67 | 543.67 | 784.54 | 551.17 | 617.17 | 567.79 |
| 11 | 1100.64 | 1189.59 | 1235.61 | 1151.23 | 1238.14 | 1243.88 | 668.67 | 561.96 | 809.43 | 567.17 | 635.38 | 589.17 |
| 12 | 1286.00 | 1167.73 | 1301.24 | 1207.19 | 1397.00 | 1396.88 | 506.83 | 413.54 | 473.41 | 423.75 | 495.17 | 426.75 |
| 13 | 1383.25 | 1205.74 | 1412.09 | 1320.63 | 1307.73 | 1262.48 | 499.71 | 401.38 | 563.09 | 384.48 | 524.83 | 391.70 |
| 14 | 1508.17 | 1273.29 | 1535.22 | 1368.96 | 1397.60 | 1478.25 | 654.00 | 478.00 | 744.21 | 513.67 | 793.75 | 540.25 |
| 15 | 1222.78 | 1168.21 | 1152.04 | 1064.30 | 1167.92 | 1158.63 | 510.96 | 555.83 | 465.09 | 485.42 | 643.67 | 434.54 |
| 16 | 1113.23 | 1013.87 | 1152.09 | 970.22 | 1037.13 | 1111.42 | 470.00 | 398.21 | 519.10 | 418.75 | 502.33 | 471.75 |
| 17 | 1542.77 | 1571.35 | 1410.05 | 1577.57 | 1542.92 | 1604.17 | 507.88 | 393.46 | 565.36 | 416.58 | 571.46 | 417.33 |
| 18 | 1439.33 | 1494.57 | 1646.48 | 1656.29 | 1550.71 | 1522.13 | 660.48 | 541.58 | 878.79 | 603.21 | 732.92 | 550.50 |
| 19 | 1148.78 | 1107.21 | 1139.48 | 1267.48 | 1188.48 | 1057.96 | 689.22 | 494.33 | 699.57 | 532.67 | 769.63 | 536.13 |
| Mean | 1357.7 | 1316.3 | 1374.4 | 1332.5 | 1363.3 | 1323.2 | 620.4 | 512.4 | 732.1 | 515.7 | 668.8 | 519.4 |
| SD | 197.4 | 215.5 | 171.9 | 200.6 | 168.1 | 191.0 | 147.1 | 105.5 | 272.2 | 102.4 | 146.8 | 91.5 |
| SEM | 45.3 | 49.4 | 39.4 | 46.0 | 38.6 | 43.8 | 33.8 | 24.2 | 62.5 | 23.5 | 33.7 | 21.0 |

| [Z(ACC)-Z(RT)]/2 | ValueConClo | ValueConFar | ValueInconClo | ValueInconFar | ValueNeuClo | ValueNeuFar | SizeConClo | SizeConFar | SizeInconClo | SizeInconFar | SizeNeuClo | SizeNeuFar |
| --- | --- | --- | --- | --- | --- | --- | --- | --- | --- | --- | --- | --- |
| 1 | -0.68 | -1.31 | -4.29 | -3.18 | -1.28 | -1.71 | 2.60 | 2.65 | 0.65 | 2.50 | 1.56 | 2.63 |
| 2 | -0.82 | 0.30 | -5.38 | -1.65 | -1.37 | 0.07 | 1.02 | -0.08 | -4.72 | 1.64 | -0.45 | 2.99 |
| 3 | -3.28 | -2.96 | -2.71 | -3.54 | -4.41 | -3.33 | 1.12 | 3.04 | 0.35 | 3.01 | -0.57 | 2.69 |
| 4 | -1.19 | -0.84 | -0.57 | -1.01 | -1.48 | -0.88 | 0.17 | 1.81 | -3.10 | 1.58 | -0.55 | 1.85 |
| 5 | -3.63 | -3.63 | -2.78 | -2.62 | -1.79 | -0.53 | 1.31 | 2.78 | -0.55 | 1.97 | 1.56 | 2.60 |
| 6 | -0.51 | -0.51 | -2.40 | -1.48 | -2.12 | -0.75 | 1.57 | 2.74 | 2.01 | 2.63 | 1.38 | 2.60 |
| 7 | -3.04 | -4.21 | -2.51 | -1.30 | -1.64 | -1.99 | 1.41 | 2.14 | 0.54 | 2.29 | 0.79 | 2.47 |
| 8 | -1.93 | -2.26 | -3.22 | -2.17 | -2.97 | -2.39 | 1.82 | 1.37 | -1.10 | 1.68 | 1.09 | 1.55 |
| 9 | -0.85 | -0.40 | -0.98 | -0.05 | -2.44 | -1.39 | 1.47 | 2.00 | 1.27 | 2.83 | 0.67 | 2.78 |
| 10 | -2.60 | -2.44 | -1.32 | -2.34 | -2.04 | -1.63 | 2.08 | 2.49 | 1.44 | 2.45 | 2.17 | 2.38 |
| 11 | -1.40 | -1.79 | -1.26 | -1.62 | -2.73 | -0.57 | 1.94 | 2.41 | -0.86 | 2.38 | 2.09 | 2.29 |
| 12 | -2.94 | -1.69 | -5.93 | -2.60 | -7.07 | -1.23 | 1.92 | 3.05 | 1.33 | 3.01 | 2.70 | 3.00 |
| 13 | -1.17 | -1.13 | -2.03 | -0.90 | -2.30 | -1.38 | 2.68 | 3.11 | 0.94 | 2.45 | 1.84 | 2.42 |
| 14 | -1.72 | -0.69 | -2.57 | -1.11 | -4.16 | -1.59 | 2.00 | 2.77 | 1.61 | 2.62 | 1.40 | 2.50 |
| 15 | -1.20 | -0.24 | -0.90 | -0.51 | -0.23 | -0.19 | 2.63 | 2.43 | 1.37 | 2.74 | 2.05 | 2.96 |
| 16 | -1.46 | -0.29 | -0.90 | -0.10 | -0.39 | 0.01 | 2.81 | 3.12 | 0.40 | 3.03 | 2.67 | 2.80 |
| 17 | -3.33 | -2.72 | -3.48 | -2.75 | -1.87 | -2.14 | 2.64 | 3.14 | 0.93 | 3.04 | 2.36 | 3.04 |
| 18 | -1.42 | -2.39 | -4.51 | -2.36 | -1.90 | -1.78 | 1.25 | 2.49 | 1.03 | 2.23 | 1.66 | 2.46 |
| 19 | -0.88 | 0.03 | -0.84 | -1.40 | -1.05 | 0.24 | 1.12 | 2.70 | 1.08 | 2.53 | 1.50 | 2.52 |
| Mean | -1.79 | -1.53 | -2.56 | -1.72 | -2.28 | -1.22 | 1.77 | 2.43 | 0.24 | 2.45 | 1.36 | 2.55 |
| SD | 1.01 | 1.29 | 1.60 | 1.00 | 1.57 | 0.94 | 0.71 | 0.77 | 1.70 | 0.47 | 1.00 | 0.38 |
| SEM | 0.23 | 0.30 | 0.37 | 0.23 | 0.36 | 0.21 | 0.16 | 0.18 | 0.39 | 0.11 | 0.23 | 0.09 |
